# Patient and public perspectives on cell and gene therapies: a systematic review

**DOI:** 10.1038/s41467-020-20096-1

**Published:** 2020-12-08

**Authors:** Olalekan Lee Aiyegbusi, Karen Macpherson, Lauren Elston, Susan Myles, Jennifer Washington, Nisha Sungum, Mark Briggs, Philip N. Newsome, Melanie J. Calvert

**Affiliations:** 1 Centre for Patient Reported Outcomes Research, Institute of Applied Health Research, University of Birmingham, Birmingham, UK; 2 National Institute for Health Research Birmingham Biomedical Research Centre, University of Birmingham, Birmingham, UK; 3 National Institute for Health Research Applied Research Centre West Midlands, and National Institute for Health Research Surgical Reconstruction and Microbiology Research Centre, University of Birmingham, Birmingham, UK; 4 Birmingham Health Partners Centre for Regulatory Science and Innovation, Birmingham, UK; 5 Healthcare Improvement Scotland, Glasgow, Scotland UK; 6 Health Technology Wales, Cardiff, UK; 7 Midlands-Wales Advanced Therapy Treatment Centre, University Hospitals Birmingham NHS Foundation Trust, Birmingham, UK; 8 Welsh Blood Service, Velindre University NHS Trust, Cardiff, UK; 9 Centre for Liver and Gastrointestinal Research, Institute of Immunology and Immunotherapy, University of Birmingham, Birmingham, UK; 10 Liver Unit, University Hospitals Birmingham NHS Foundation Trust, Birmingham, UK

**Keywords:** Medical research, Scientific community

## Abstract

Cell and gene therapies offer opportunities for treating disease with potential to restore function, and cure disease. However, they are not without risk and pose complex logistical, economic, ethical and social challenges for health systems. Here we report our systematic review of the current evidence on patient and public knowledge and perspectives of cell and gene therapies, to inform future research, education and awareness raising activities. We screened 10,735 titles and abstracts, and evaluated the full texts of 151 publications. The final selection was 35 publications. Four themes were generated from the narrative synthesis of the study findings namely: (1) Knowledge and understanding of cell and gene therapies, (2) Acceptance of cell and gene therapies (3) Understanding of risk and benefits of therapy, and (4) Information needs and current sources of information. As potential funders or future recipients, it is important that the public and patients are aware of these therapies, understand the issues involved, and can contribute to the debate. This review highlights the need for appropriate patient and public education on the various aspects of cell and gene therapies. High quality studies exploring patient and public opinions and experiences of cell and gene therapy are required. Patient and public perceptions of these therapies, alongside evidence of clinical and cost-effectiveness, will be central to their uptake and use.

## Abstract (web summary)

Cell and gene therapies offer opportunities for treating disease. Here, the authors conduct a systematic review of the evidence of patient and public perceptions of gene and cell therapies, and highlight the need for appropriate patient and public education, noting that patient perception, alongside evidence of clinical and cost-effectiveness, will be central to uptake and use of these therapies.

## Introduction

Over the last decade, new cell, gene and tissue-engineered therapies have been developed to treat various cancers, inherited diseases and some chronic conditions^1^. They offer opportunities for the treatment of disease and injury, to restore function, and in some cases offer cures^1–4^.

These therapies are approved and regulated in the US by the Center for Biologics Evaluation and Research^5^ and in Europe by the European Medicines Agency (EMA)^6^. The EMA refers to cell and gene therapies along with tissue-engineered medicines as Advanced Therapy Medicinal Products (ATMPs)^7^.

The provision of these therapies present logistical and delivery challenges for health systems globally^8^. In addition, there are complex social, ethical, health and economic issues to navigate which require the input of patients, carers and the public to ensure successful resolution^9,10^. A high level of engagement, awareness and understanding among patients and the public will ensure their vital contribution to policy debates and enhance their ability to make informed decisions about participation in trials and routine administration of cell and gene therapies^11^. It is therefore crucial that health systems, regulatory bodies and researchers are aware of the knowledge and perspectives of patients and the public as this insight may guide information provision, patient and public involvement (PPI) activities and inform the development and delivery of targeted educational interventions.

In this work, utilising systematic approaches, we summarise and discuss the current evidence on patient and public opinions on cell and gene therapies: their acceptance of these therapies, their understanding of the associated risks and benefits, and their views regarding reimbursement. In addition, we explore the potential influences on their views and highlight future priority areas for research. We focus on therapies we anticipate will gain increased licensing within the next decade. For this reason, we exclude studies that dealt solely with embryonic stem-cell therapy or germline gene therapy due to the complex legal and ethical issues associated with these therapies. We provide descriptions of the cell, gene and tissue-engineered therapies in the Supplementary Table 1.

## Results

The searches returned 11,786 bibliographic records. After removing 1051 duplicates, 10,735 titles and abstracts were screened. Of these, 151 articles were taken forward and following full-text review, 33 papers were selected. Two publications were identified following a hand search of reference lists giving a total of 35 studies (Supplementary Fig. 1). The article selections by K.M. and O.L.A. fully matched at the screening stage. At the full-text review, the reviewers had to discuss 5% of the articles further before making a final decision.

Eighty-two per cent of the included articles were published between 2012 and 2020. Of these, 62% were published between 2015 and 2020. Twenty-four of these papers related specifically to cell therapy while 11 pertained to gene therapy. Five of the included studies were conducted in the UK^12–16^, eight in the USA^17–24^, six in Canada^25–30^, two were conducted in Australia^31,32^ and one each from Belgium^33^, China^34^, Germany^35^, Hungary^36^, Ireland^37^, Japan^38^, Korea^39^, South Korea^40^ and Sweden^41^. One study included multiple countries across Europe^42^, three studies were set in Canada and USA^43–45^ and one included Europe, USA and Canada^46^. Supplementary Table 4 provides further details on the characteristics of these studies and their critical appraisal. Below, we present the four themes generated from the narrative synthesis of the study findings. Table 1 details key areas for the patient and public education based on the findings of this review.

**Table 1:** Key areas for patients and public education.

Risk of infection from donated genes/cells, viral vectors and the sources of cells	These important topics require the provision of accurate and adequate information as a number of the included studies indicated that the perceived risks of infection and the source of stem cells might influence patient and public attitudes towards cell and gene therapies. These attitudes may in turn influence political decisions; determine the success of trial enrolment and retention as well as the subsequent adoption of cell and gene therapies.
Expectations of benefit from therapy and understanding of treatment risk	Overestimation of therapeutic benefits or underestimate risks of harm also known as “therapeutic misestimation” may be addressed by ensuring that the information provided is clear, balanced and unbiased. Especially as media discourse tend to focus on sensational aspects of cell and gene therapy and online media sources, in particular, are often less critical in their reports^44^ thus potentially fuelling therapeutic misestimation. The findings on conflict burden experienced by carers indicate the need to be aware of the complex and at times, conflicting caring and interpersonal contexts which may influence the decision-making process.
Engagement with clinicians	The finding that some patients avoid discussing their interest in cell and gene therapies with their physicians^43^ may signify that some already have the notion that physicians may downplay or dismiss potential benefits. Therefore, whilst managing unrealistic expectations is important, it is also essential that the potential benefits are not downplayed as this may risk eroding the trust patients and members of the public have in the healthcare system.
Time frames for translation of products from research to clinical application	Overoptimistic messages about time frames may not only lead to disappointment but also undermine trust in researchers and threaten funding support^25^. Information on the processes involved in the translation of research to the clinical application should be provided in lay terms. This may facilitate a reassessment of patient expectations in terms of participation in clinical trials and the availability of therapies in a clinical setting in light of patient-specific therapeutic windows.
Clinical research versus routine clinical application	There is a need for patients and members of the public to understand the distinction between clinical trials and routine clinical application. Clarifications are also required about the aims of each clinical trial phase as the review findings suggest that there are considerable therapeutic misconceptions among patients and members of the public.
Unlicensed versus licensed indications for cell and gene therapies	Although not the focus of our review, it is important that patients are aware of the health risks of “stem-cell tourism”, which often involve treatment with unlicensed therapies. Patients rarely see the warnings on unproven therapies posted on regulatory websites. Such information needs to be provided through a combination of channels patients use and trust including their physicians^7^.

### Knowledge and understanding of cell and gene therapies

The studies reported varying levels of patient knowledge and understanding of cell and gene therapies^13,19,23,31,33,40,41^. Studies in patients with ischaemic stroke, cystic fibrosis and sickle cell anaemia found that they had limited prior knowledge of cell and gene therapy^13,23,40,41^. Participants with human immunodeficiency virus (HIV), in a study, were aware of the potential application of gene therapy in conditions like Parkinson’s disease, yet few knew about its potential use in HIV/AIDS treatment^19^. A study conducted with a few young adults reported high levels of awareness of gene therapy^35^. The male gender and higher educational degrees were also associated with a higher level of knowledge on cell therapies in a mixed population of cancer patients and members of the public^33^.

In general, patients were uncertain about the sources of cells, the use of viral vectors and their likelihood of transmitting infection, and the risks of concomitant chemotherapy^19,23^. They were also unclear about the distinction between the use of the therapies in research and their clinical application as licensed therapies^36^.

Patients often struggled to understand the reason for the prolonged time interval between scientific discoveries and the commencement of trials and subsequent regulatory approvals. They often worried that the time frames may not match their own limited therapeutic window especially as their conditions deteriorated^21,25^.

There were “therapeutic misconceptions” among patients where they confused the goals of research and those of clinical care^25^. Many also appeared to confuse the goals of various phases of clinical trials and did not realise that the main focus of phase 1 trials is to establish safety and not demonstrate therapeutic benefit.

Regardless of their level of knowledge, patients generally expressed a desire for more information and clarity^19,21,23,32,43^. However, some patients were of the opinion that they did not need to fully understand the science behind these therapies, trusting the opinions of the healthcare providers on efficacy and safety^13^.

The studies with members of the public focused on opinions rather than actual knowledge of cell and gene therapy. However, based on some of the studies, it could be inferred that just like patients, the public do not perceive themselves well informed on the subject and desire more information^34,38^. A study also reported differences in the type of information the public desire and that which is of interest to the scientific community^38^. For instance, members of the public were more interested in the cost of therapies and measures for safety^38^.

### Acceptance of cell and gene therapies

Acceptance of cell and gene therapies varied among patients^15,17,19,21,23,31,41,43^ but generally increased after the provision of information^19,21,23,41^. There was a tendency for respondents who were more accepting to focus on potential benefits that may be gained overtime or the possibility that new treatments may become available before the potential benefit from gene therapy ended^21^. Male gender, older respondents, higher education, longer duration and greater severity of the underlying condition and greater risk of death were associated with greater acceptance of stem-cell research and the perception that it is beneficial for the society and morally acceptable^15,21,23,33,39^. There were also indications that older patients may have altruistic motivations for participating in gene therapy trials^23^. One study explored the issue of cost by asking stroke patients how much they were willing to pay for cell therapy. The majority (67%) felt a reasonable price would be under US$1000 at a time when the actual cost of treatment was estimated as ranging from US$5000 to $39,500^40^.

The source of stem cells influenced attitudes towards cell therapies and was at times a source of confusion among patients^19^. There were very limited reports of the explorations of ethics in the articles focused on patient and carer perspectives. Some participants in the study by Wright et al. commented that more efforts should be made to prevent diseases rather than finding a cure^15^. The use of the HIV vector in gene therapy was of concern to participants in the study by Strong et al.^23^. Whilst it was uncertain that the provision of clarification about the use of the HIV vector changed patients’ opinion of gene therapy, they suggested that educational materials should emphasise that the virus is not present in the therapy otherwise patients may be put off^23^.

Studies involving the public generally reported support for cell and gene therapy research^16–18,24,29,30,34,38,42,45,46^ with most participants in some studies confirming that they would be happy to donate their tissue for research^14,17,22,38^. A study showed that in the West, support for stem-cell research might vary by region with the highest level found among Canadians, then Americans and finally Europeans^46^. Public support for cell and gene therapies was highest for potentially fatal or severely debilitating diseases^34,45^. Similar to the patient findings, men and individuals with higher education were more likely to support cell and gene therapy^18,42^. In contrast, older people were found to be less supportive^42^.

Public opinions about the ethics of cell and gene therapies were explored by a number of studies^18,28,30,42,45^. These discussions focused on research into the use of embryonic cells and gene editing for non-therapeutic purposes. Majority of the studies reported low public acceptance for these types of cell and gene research^18,30,42,45^. The use of embryonic cells was generally perceived as amoral, ‘crossing the line’ and ‘playing God’^18,30,42,45^. There were concerns that gene editing for human enhancement may threaten genetic diversity in the long term^30^. There were also concerns that if cell and gene therapies become freely available, governments may interfere with the individual/parent’s right to choose and make it mandatory to undergo such treatments^30^.

Among individuals that supported the use of gene editing for enhancement purposes, the highest levels of support were for increasing life span, improving intelligence, and improving strength and fitness^45^. A neutral response to the use of gene editing for enhancement was reported in the Chinese population^34^. Individuals with children were reported to be more likely to be in support^34^. Interestingly, about 60% of the participants in a study expressed support for the use of embryonic stem cells with the proviso that strict government regulations are in place^28^. The influence of religion on public beliefs were generally inconclusive^45^.

There were discussions about the cost of treatment and funding for cell and gene research. The high cost of treatment was a concern to the Chinese public^34^ and a study of online posts reflected public concerns over limited availability and central funding of stem-cell therapy^44^. In fact, a US poll reported a consistent trend suggesting public support for the easing of government restrictions on stem-cell research funding^20^. Some participants expressed support for greater scientific freedom which could lead to therapeutic discoveries. On the other hand, some feared scientific malpractices may occur if restrictions were relaxed^17,28^ while others were worried about the adequacy of regulatory approval system.^42^

### Understanding of risk and benefits of therapy

In a number of studies, patients tended to overestimate the benefits of therapy, particularly around whether the therapies would be disease limiting or disease reversing^12,21,33,39,40^. Some studies reported that patients were so eager to gain access to therapy, that they expressed minimal consideration for the potential side effects and had unrealistic expectations around the timescales for the availability of actual therapeutic options^21,25,33,35,39^. These patients tended to be male, older respondents with longer duration and greater severity of underlying condition^21,33,39,40^.

Conversely, some patients were worried about the considerable uncertainty around the benefit that may be derived given the potential risks of gene therapy^21,23^. A study reported that parents of younger children, in particular, expressed more concerns about the benefit/risk balance^21^. However, the majority of the parents in this study confirmed that their tolerance for risk would increase as their children grew older, the child’s health deteriorates, and treatment options become more limited^21^. Some adults with poor health status also shared similar views^21^. There were also concerns about the use of chemotherapy in the gene therapy^23^. Given the association of chemotherapy with cancer treatment and the risk of developing cancer, patients were worried the side effects of chemotherapy would be taxing on their bodies and they could be trading their current illness for cancer^23^. The participants were of the opinion that more information about potential benefits, risks and logistical requirements of participating in trials is required to make an informed assessment of benefit versus risk^23^.

A study reported that partners and carers of patients with stroke often experienced ‘conflict burden’^12^. They strived to balance their concerns about risks with the hope that their loved ones’ condition would improve by participating in clinical trials^12^. The carers reported a sense of guilt for holding reservations and not being as supportive as they felt they ought to when the patients nursed high hopes of recovery. In addition, there were concerns that patients might have difficulties understanding trial information, due to cognitive impairments, during the acute post-stroke period, leading to poor decisions^12^.

### Information needs and current sources of information

Patients frequently indicated a desire for more information regardless of their gender, age or educational status^19,23,32,43^. Some patients suggested that educational materials should report results from large, long-term studies and explicitly report the risks and side effects observed in these studies^23^. They also expressed a need for more information on the eligibility criteria for studies and more personalised information^21,23^.

Television was identified as the major source of information on cell and gene therapy for patients, along with newspapers and magazines, the radio, clinicians, friends and colleagues^40,41^. However, the study by Aked et al., reported that patients perceived physicians as the most reliable source of information^41^. It was interesting to note that despite the view that information obtained from physicians was more trustworthy, many patients may not discuss cell and gene therapy with their physicians^43^. Patients identified facilitators of trusted communications between themselves and physicians to be caring, attentive and positive physician attitudes and the patients’ positive perceptions of physician knowledge^43^. Some participants in search of guidance expressed frustration at the attitude of professionals who adopted a non-committal stance or provided inconsistent and conflicting advice^32^.

Public trust in the information provided by scientists, medical researchers and ethicists was reported to be substantially higher than trust in any other social group including the media, religious leaders and the US political system^20^. There was also distrust of regulatory authorities in Central and Eastern Europe^42^. Media coverage was often considered sensational, mostly reporting specific breakthroughs or safety incidents^26,36^. Articles on embryonic stem cells dominated newspaper reports on stem-cell therapy for over a decade (1998–2010) and often positioned induced pluripotent stem cells as an ‘ethical’ alternative when these were discovered in 2007^26^. There was a tendency for online media coverage to omit information about the existence or lack of evidence to support cell and gene therapies and the potential risks^44^.

### Hypothetical relationships between the themes

**Fig. 1:**
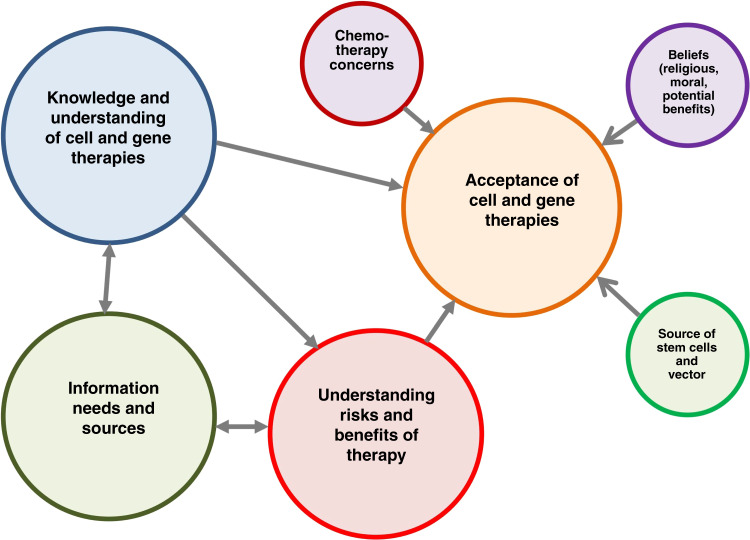
Hypothetical model of thematic relationships based on review findings. Arrows indicate hypothetical unidirectional relationships. Double arrows indicate hypothetical bidirectional relationships.

Figure 1 shows a hypothetical model of the inter-relationships between the various themes and sub-themes identified in our review. Based on our findings we hypothesise that the acceptance of cell and gene therapy by patients and the public is influenced by their knowledge and understanding of these therapies; their understanding of the potential risks and benefits; and concerns which may be procedural, ethical or religious. Their knowledge and understanding of the risks and benefits are in turn influenced by the information they receive from various sources and its trustworthiness. Whilst our review also found relationships between the acceptance of cell and gene therapies and issues such as the use of chemotherapy and viral vectors in gene therapy procedure^23^, and beliefs; it is possible that other relationships exist which were not captured by the included studies.

### Critical appraisal

*Response rates*: Of the 25 publications expected to report response rates (studies reviewing or re-analysing data may not have access to such data) only 10 (40%) did. Reported response rates were high (>75%) in five studies^15,31,40,41,47^, moderate in (50–60%) in two^23,35^ and low in three studies (<30%)^13,29,38^.

*Sample sizes*: The sample size used in a study has an impact on the precision of its findings. Sample size requirements were expected to vary depending on the study methods. A few studies reported sample sizes smaller than the recommendations for surveys^15,41^ and qualitative research^13,21,27,35^. However, this should be interpreted with caution as three of the qualitative studies were conducted with patients with rare diseases (cystic fibrosis^13^, RPE65 deficiency^35^ and Duchenne muscular dystrophy^21^). These studies often struggle with patient recruitment due to very small patient populations.

*Participant characteristics*: Information on participant characteristics assist with the determination of potential recruitment bias and the generalisability of the findings. There was a wide variation in the level of reporting of participant characteristics. Understandably, studies relating to the media, re-analysis of poll data and rare diseases might not be able to report this in detail. In addition, studies in rare diseases often provide limited information on patient characteristics in order to preserve patient anonymity.

*Study methods*: Various issues were identified in the methods used by the authors. For instance, Benjaminy et al.^25^ and Peay et al.^21^ recruited patients via patient advocacy groups which might indicate potential recruitment bias. However, Peay et al. argued that the advocacy group reflected the patient population and trial participants would most likely be recruited through these organisations.

*Funding and competing interests*: This was considered an important indicator of study quality as it assists with the assessment of the potential influence of the funder on the work and the validity of study findings. The source of funding was reported by 83% of the publications. Majority of which were non-pharmaceutical. Most of the publications indicated that funders had no role in the conduct of the studies. However, Peay et al.^21^ declared that although funders were not involved in data collection and analysis, they were involved in the interpretation and generation of conclusions. It was impossible to assess to what extent the funder influenced these aspects of the work. Finally, only 60% of the articles provided an author statement of competing interests. Further details of the critical appraisal of the included studies can be found in Supplementary Table 4.

## Discussion

Acceptance of cell and gene therapies varied among patients but generally increased after the provision of information. Male gender, older respondents, higher education, longer duration and greater severity of the underlying condition and greater risk of death tended to be associated with greater acceptance of stem-cell research among patients. Members of the public generally expressed acceptance of cell and gene therapy with some geographical variation. A number of the studies reported that participants frequently indicated a desire for more information regardless of their gender, age or educational status. They were generally of the opinion that more information about potential benefits and risks of participating in trials is required to make an informed assessment of benefit versus risk. Therapeutic misconceptions and misestimations were common among patients and members of the public. Whilst physicians were generally regarded as the most trustworthy source of information by patients, some never discussed cell and gene therapies with their physician while some expressed frustration at the ambivalence of their physician. A few studies explored public trust in information sources of cell therapies. Whilst medical professionals and scientists again ranked highest as the most trustworthy, politicians and governmental institutions were among the lowest ranking. Our review found that there are ongoing debates globally on the ethics of research into and the use of these therapies. These debates were especially strident when research involving the use of embryonic cells and gene editing was discussed. The issue of ethics was linked to equity in access to treatment, allocation of government funding to support research into cell and gene therapies and reimbursement for products available to treat patients.

We compared our findings to those from oncology trials where traditional modalities of chemotherapy and radiotherapy are used. The issue of therapeutic misconception among some patients as highlighted in our review seemed to also occur in these settings as a study reported that only 33% of respondents were actually clear about the purpose of phase 1 oncology trials and 85% decide to participate in phase 1 trials expecting significant therapeutic benefits^48^. Similar to our review, some patients with advanced cancer were found to overestimate not only durability but also the chance of tumour response, symptom palliation and survival benefit^49^. The enthusiasm of other stakeholders, with various personal goals, such as researchers, politicians and funders in promoting optimistic timelines often conceal the fact that the process of translating scientific breakthroughs to clinical applications takes time and may further compound the issue of therapeutic overestimation among patients and members of the public.

Although public opinions on gene editing in babies were not the focus of our study, there were references to it by members of the public. We, therefore, compared our findings with the results of the polls conducted by *Stat* and Harvard T.H. Chan School of Public Health and the Pew Research Center on the subject^50,51^. There were broad similarities in public views on issues such as a general lack of support for gene editing to reduce the risk of developing serious diseases and very low support for human enhancement purposes^52^. The issue of federal funding for gene therapy research received a high level of support across the polls with a significant number of respondents (44%) in the STAT-Harvard poll supporting federal government funding of gene editing in babies to reduce their risk of developing serious diseases^50^. Another interesting finding in the STAT-Harvard poll was that 53% of respondents felt scientists and physicians should make the decision on gene editing. Only 9% felt it should be up to the government and policymakers to decide^50^. These reports echo our finding that members of the public tended to trust scientists and physicians significantly more than politicians and governmental organisations. The implication of this finding is that physicians and scientists are best placed to provide accurate information that will be trusted by patients and the public. It is therefore essential that they are actively engaged in the entire process of developing cell and gene therapies. However, these professionals need to be aware of the reputational risks involved and must be transparent about potential conflicts of interests.

Comparing the views and opinions reported in the included articles published earlier to those expressed in the more recent ones, there were no radical differences in opinions and it appeared that whilst the field of cell and gene therapy is making rapid scientific advances, patient and public perceptions are not changing as fast. This observation was supported by the report by researchers at Pew Research Center that the findings in their 2018 survey on public attitudes towards genetic editing were consistent with findings from their 2014 and 2016 surveys^51^.

There is a possibility, however, that public opinion (and the influence of patient advocacy organisations) over time, whether accurate or not, has influenced political position on stem-cell therapy which in turn has influenced policy, research funding and regulation. For instance, the decision by President Obama to expand federal funding for embryonic stem-cell research in 2009 was linked to the consistent trend in the Gallup polls which showed that majority of American respondents would like the government to ease restrictions on stem-cell research funding^20^. It is worth noting that the President himself cited public support as a factor for making the decision^53^. It is, therefore, essential that up-to-date and accurate information be provided to patients and members of the public in the first place given their potential influence on political decisions. The perceptions and support of patients and members of the public have been shown to be critical for the adoption of cell therapies by the healthcare sector and a lack of such support may significantly hamper development and adoption^54^. This further underscores the need to assess existing opinions among patients and members of the public and then develop appropriate and trustworthy strategies to correct misconceptions and provide unbiased current information.

The mistrust of the information provided by the government may be an extension of the general mistrust of political systems or a perception that governmental position is often swayed by party politics and not entirely dependent on scientific facts even though as shown earlier public opinion might be a strong influence itself. It is possible that an awareness of this public mistrust led to the UK government including a statement about trust in its recent White Paper on the regulation of new technologies “… *We need to build trust and enable both consumers to have confidence in innovations and businesses to have confidence in our stable and proportionate regulatory system”*^55^.

Whilst the media was ranked low in terms of trustworthiness, its often over-optimistic and unbalanced portrayals of the potential benefits of cell and gene therapy undoubtedly influence the perceptions of patients and members of the public and fuel therapeutic overestimation^56–58^. It was interesting to find that whilst social media has often been considered as the most popular source of information in recent years, a number of patients still considered traditional media such as the television as their primary source of information on cell and gene therapy^40,41^. Aked et al.^41^ reported that most of the respondents had more than one source of information and we suspect that this would be the case for most individuals.

It was proposed by Allum et al.^46^ that at least two key dimensions - moral concerns and beliefs about benefits, frame public views on stem-cell research. We observed from our review of the literature, that whilst there was a tendency for members of the public to consider and express their opinions about the ethics of cell and gene therapy, this was less frequently raised by patients. This might be due in part to the line of questioning by interviewers. However, we hypothesise that patient views are mostly framed by the potential risks and benefits of cell and gene therapy (i.e. the risk-benefit ratio) with ethical concerns carrying less weight in the discourse. It is essential that this potential difference in how issues are prioritised by both groups is considered and guides the design of any educational interventions. This also shows the importance of tailoring any educational intervention to the needs and concerns of the particular audience.

Patients and members of the public are generally unaware of the timescales and challenges manufacturers face developing cell therapies; obtaining regulatory approvals; scaling up production utilises costly technological resources to ensure the cells remain potent throughout the production process and storing and delivering the cells to the patient and physician in a viable and functional condition^54^. Developing cell and gene therapies is a costly venture and securing reasonable reimbursement is an important post-market challenge^54^. Reimbursement needs are at a level higher than the cost of production otherwise the venture is not viable. The question about whether the individual or the government should pay for the therapy is central to this issue. If this falls to individual patients, a significant number of patients will be unable to purchase such expensive therapies leading to inequity in access. It has to be said that some patients already pay for their treatment sometimes travelling abroad to obtain therapies unlicensed in their home countries, the so-called ‘stem-cell tourism’. In the US, due to gaps in regulation, a number of biotechnology companies were reported to operate on a pay-to-participate basis and require patients to pay before they were enroled to clinical studies^22^. The most equitable scenario is government funding but this in the long term may be uncertain as funding policies may change. It is, therefore, no surprise that many biotechnology companies have crashed and burned over the years^54^.

We did not identify any studies that examined the experiences of people who have received cell or gene therapies. There is, therefore, a need to capture the experiences of such patients in future to improve the evidence base. As therapies start to be considered by health providers such as the NHS and US Medicare/Medicaid, which place value on including the perspectives of patients, such information may start to become more widely available. None of the studies specifically considered patient views on reimbursement, prioritisation or geographical variation in the provision of cell and gene therapies. Future studies need to address these issues in depth. The role of the media in information provision, especially with the increasing use of social media, needs to be explored further. The need for a balanced presentation of information is important. Strategies to ensure that the media serves as a trustworthy source of information need to be developed.

We found fewer articles on gene therapy compared to those on cell therapy. Whilst this might mean that our review does not completely capture patient and public views of gene therapy, we believe it is useful to report what we found. This imbalance in the number of articles retrieved highlights the fact that more research on perspectives of gene therapy is needed. This study relied on the information provided by the included publications, which were sometimes limited in detail and quality. Questions posed to participants were framed in different ways (open-ended and or closed-ended). For instance, the survey studies employed close-ended questions that do not reflect emergent opinions or reflect participant-driven concerns. They have also been reported to possess low validity and carry the risk of producing framing effects. For example, the act of asking whether an issue is of ethical concern may imply that it is^30^. It was impossible for us to assess or measure the effects of these methodological issues based on the information reported by the authors. In addition, virtually all the studies reported providing participants with information sheets and/or verbal information prior to obtaining informed consent. It is impossible for us to ascertain how much information about cell and gene therapy was provided, and to what degree this influenced participants’ responses during the studies. The included studies were of variable quality based on our critical appraisal with methodological issues the most prevalent. However, it is possible that some of this is due to a reporting approach where the provision of details on results and its interpretation in the discussion sections is prioritised over providing essential details on methodology. The studies included in this review utilised a variety of methods including interviews, focus groups and surveys to elicit patient and public views. There was an absence of papers using consensus methods in the selected studies. Modified Delphi technique, nominal group technique or other consensus-based methods may be used to reach consensus on priority areas for education and information provision, ideally through discussion with other key stakeholders such as clinicians, ethicists, regulators, industry and policymakers. For those interested in valuing patient/public preference for alternative therapies (e.g. the use of highly expensive but potentially curative treatments for a limited number of patients versus cheaper but non-curative treatments for a larger number of patients) there are specific health economic techniques such as stated preference methods, which could be used. These techniques, however, are time-consuming and resource-intensive. This review highlights the need for high-quality studies exploring patient and public opinions of cell and gene therapy. We recommend that future studies follow current methodological guidance such as the consolidated criteria for reporting qualitative research^59^ and the guideline for survey research^60^, which can be found on the Equator Network website.

Although not the focus of the present study, PPI in research is an important and vital component of patient-centred care and clinical research. PPI has been defined as “…research being carried out ‘with’ or ‘by’ patients and members of the public rather than ‘to’, ‘about’ or ‘for’ them”^61^. PPI has been recognised as a vital activity that may facilitate the acceptability, relevance, timeliness and quality of research^62–64^. PPI may occur at an organisational level where individual patients and members of the public are involved in the development of cell and gene therapy by providing feedback and collaborating at all stages of clinical trials, dissemination of trial results and finally involvement in decisions regarding licensing reimbursement of cell and gene therapies. It could also occur at a higher level where patient organisations, exert advocacy influence. For instance, ensuring that research into stem-cell therapies receives appropriate funding from the government. We noted minimal reports of PPI in the included studies and recommend that patients should be involved in the co-design of future studies. The expectation is that the findings of this review may also inform the design of future PPI initiatives in the field of cell and gene therapy.

Cell and gene therapies have the potential to change the treatment of diseases, which currently have limited treatment options. However, there are associated risks to patients and complex challenges translating these scientific breakthroughs into clinical applications. Patient and public perceptions could play a vital role in the development of therapies and influence their subsequent uptake. Patients and carers need to be provided adequate and accurate information which may influence their perception and acceptance of cell and gene therapy. They need to be provided information on the potential benefits that may be gained from cell and gene therapy and the risks involved so that they can make informed decisions about participation in clinical trials and licensed routine administration. Working with patient partners in the co-design of further research and or educational resources is central to addressing the issues we have highlighted.

## Methods

The protocol for this review was registered with PROSPERO (Ref.: CRD42019131831). The study was reported according to the Preferred Reporting Items for Systematic Reviews and Meta-Analyses (PRISMA) checklist^65^.

### Search strategy

We adapted the Scottish Intercollegiate Guidelines Network (SIGN) Patient Issues filter to search for relevant research studies (https://www.sign.ac.uk/search-filters.html) (Supplementary Table 2). There were no language or study design restrictions. The following databases were searched:

- Medline
- Embase
- CINAHL
- PsychINFO
- Cochrane Database of Systematic Reviews
- DARE
- HTA
- Cochrane Central Register of Controlled Trials, Current Controlled Trials and the PROSPERO databases were searched to identify any ongoing studies, reviews and grey literature as recommended by Blackhall and Ker^66^.

The searches were conducted between 4th December 2018 and 5th April 2019 and restricted to studies published from the 1st January 2009 onwards. The cut-off date of 2009 was selected because the EU legislation on ATMPs (Regulation (EC) No 1394/2007) came into force at the end of 2008 and the EU ATMP definitions were updated in 2009 (Commission Directive 2009/120/EC). In addition, the first successful phase III gene therapy clinical trial in the EU was reported in 2009 thus signalling significant progress in the development of cell and gene therapy for clinical application^1^. We believe this date range would ensure that we capture the most recent literature whilst minimising the chance of missing any relevant studies. The search string for Medline was adapted for the other databases (Supplementary Table 2). Following peer-review, we also searched (without date restrictions) the Pew Research Center database using the ‘science’ filter (https://www.pewresearch.org/) and the entire Harvard Opinion Research Programme database (https://www.hsph.harvard.edu/horp/) to ensure that we captured any other relevant grey literature.

### Inclusion criteria

- Qualitative and or quantitative studies focusing on cell and somatic gene therapies in the clinical application or being currently considered within clinical trials.
- Studies with patients who have received, or may be eligible to receive cell or gene therapy; family carers; or members of the general public.
- Studies exploring or evaluating levels of knowledge, understanding and awareness; expectations or hopes; experiences of treatment; views, attitudes and perspectives on cell and gene therapies.

### Exclusion criteria

- Narrative reviews, commentaries, opinion pieces and letters that do not report primary findings.
- Studies focused exclusively on the perspectives of healthcare professionals and or scientists.
- Studies focused entirely on embryonic stem-cell therapy or germline gene therapy (due to the complex legal and ethical issues associated with these therapies).
- Studies reporting hematopoietic stem-cell transplants (as this type of treatment is no longer novel)^67^.

### Selection process

Search records were exported to EndNote. An experienced reviewer (K.M.) screened all the titles and abstracts. To check that the eligibility criteria had been consistently applied, another experienced reviewer (O.L.A.) independently screened 20% of the titles and abstracts. Full-text articles were retrieved for studies potentially eligible for inclusion and the same reviewers independently evaluated all articles. Reasons for exclusion were documented. Hand searching of reference lists and citation searching of the included articles was conducted. The reviewers through discussion resolved discrepancies at all stages.

### Data extraction

A data extraction form was designed and piloted by K.M. and O.L.A. on a few studies before commencing full data extraction (Supplementary Table 3). Data extracted included: (i) participant group studied and where available their age, gender, socioeconomic status and level of education; (ii) number of participants; (iii) current health of participants; (iv) type of therapy; (v) standard care; (vi) healthcare setting; (vii) study design; (viii) results, (ix) study quality and (x) author conclusions. The included papers were split between K.M., O.L.A., L.E. and S.M. for extraction. All extracted data were double-checked by another individual among the four to ensure accuracy and consistency.

### Critical appraisal

A formal quality assessment of included articles using a checklist was not conducted because the studies employed a variety of quantitative and qualitative methods which produced results that were not directly comparable. Therefore, we critically appraised each article using five quality indicators extracted using the data extraction form: (i) response rate, (ii) sample size, (iii) participant characteristics, (iv) study methods and (iv) funding and competing interests) as reported by Kirkby et al.^68^.

### Synthesis of studies

Given the high level of heterogeneity across the included studies, we did not conduct a meta-analysis of quantitative studies or a meta-synthesis of the qualitative studies. Instead, we undertook a narrative synthesis using the method described by Popay et al.^69^. This entailed: (i) A descriptive summary of the information extracted on study characteristics and critical appraisal. (ii) The exploration of associations between study characteristics and reported findings within individual studies, as well as across studies. (iii) Discussion of the findings and provision of recommendations for future research and clinical practice.

### Reporting summary

Further information on research design is available in the Nature Research Reporting Summary linked to this article.

## Supplementary information

Supplementary information

Peer Review File

Reporting Summary

## Data Availability

The authors declare that all data generated or analysed during this study are included in this published article and in Supplementary Table 4. All the publications included in this systematic review are available through open access or personal or institutional journal subscriptions.
