# Supplementary material for: Patient and public perspectives on cell and gene therapies: a systematic review
Source: Nat Commun. 2020 Dec 8;11:6265. doi: 10.1038/s41467-020-20096-1 (PMC7722871; doi:10.1038/s41467-020-20096-1)
Supplement: Supplementary file 1 — Supplementary information [file 41467_2020_20096_MOESM1_ESM.pdf]

Patient and public perspectives on cell and gene therapies: a systematic review

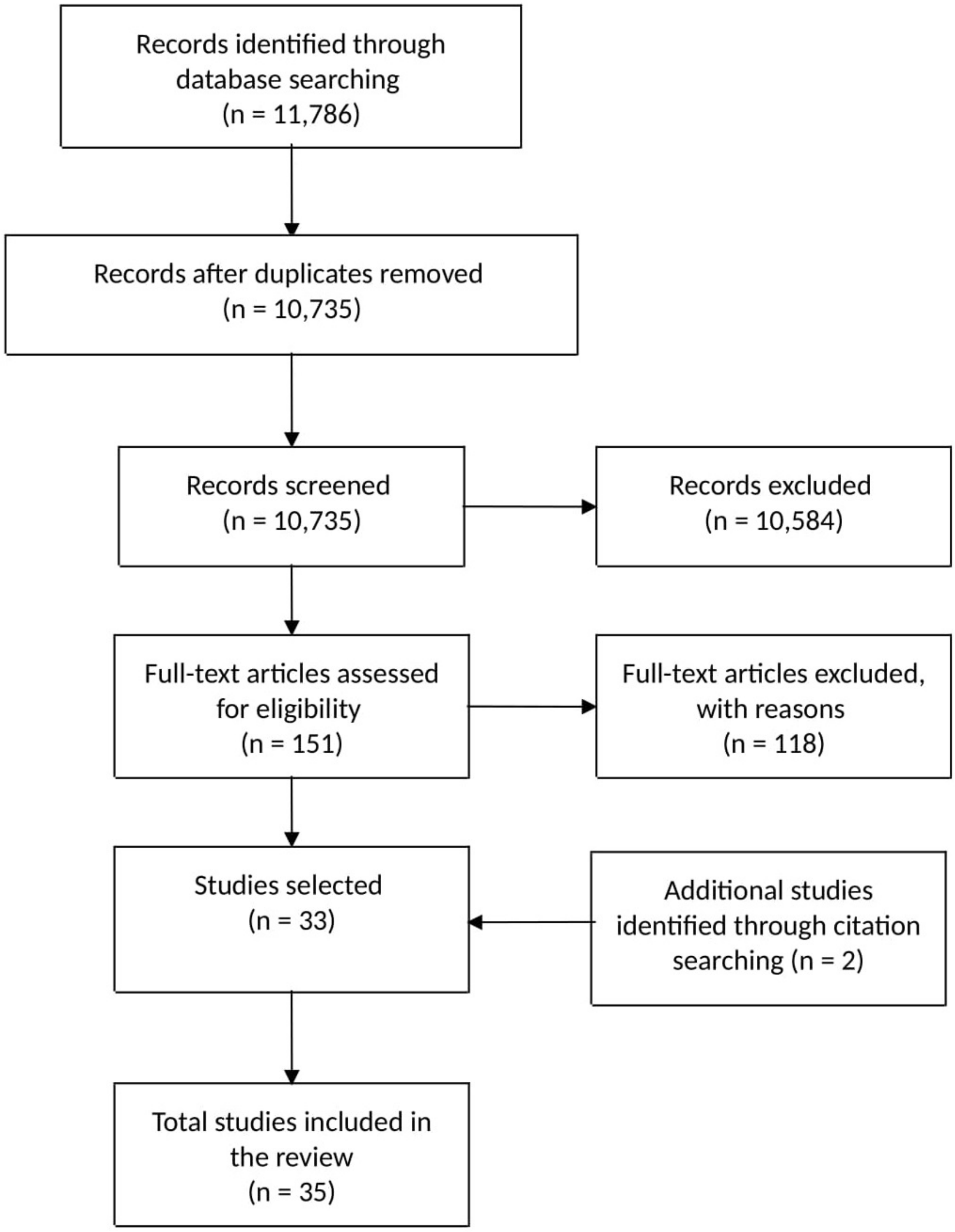

Supplementary Figure 1. PRISMA flow diagram

Supplementary Table 1. Descriptions of cell and gene therapies	
**Gene therapy medicines**	These therapies involve the replacement of damaged genes, introduction of new genes or the inactivation of mutated genes.[1] A vector, usually a modified virus which has lost its pathogenic ability, is used as a vehicle to insert a new gene into the target cells. Viruses are often used because of their ability to incorporate foreign genetic material with the host cell genome. Once inside the cells, the gene has to be 'switched on' before it functions and leads to the production of the required therapeutic protein which results in the treatment of the disease (See Figure 1). In some diseases such as sickle cell disease, it is crucial that the old stem cells with the defective gene are eliminated prior to the administration of genetically modified autologous stem cells.
**Somatic-cell therapy medicines**	These contain modified cells or tissues with altered biological characteristics, or cells or tissues not intended to be used for the same essential functions in the body.[2] These cells may be donated by another individual (allogeneic), or derived from the patient's own tissue (autologous) or from another species (xenogeneic). Autologous somatic cell therapy has the lowest possibility of triggering immune reactions which may be life threatening.
**Tissue-engineered medicines**	These contain modified cells or tissues that can be used to repair, regenerate or replace human tissue.[2] The technologies combine human tissue or cells (viable or non-viable, allogeneic or autologous) with synthetic biomaterials and may provide benefits to patients in terms of longevity, biocompatibility, and performance.[3] The most widespread existing application is skin systems for treatment of conditions such as chronic wound healing in diabetic patients with ulcers; burns; and cancer.[3]

**Supplementary Table 2. Medline search**	
**Cell and gene therapy**	
1	exp "Cell- and Tissue-Based Therapy"/
2	exp Genetic Therapy/
3	Regenerative Medicine/
4	Tissue Engineering/
5	exp Gene Transfer Techniques/
6	exp stem cells/
7	exp Multipotent Stem Cells/
8	Induced Pluripotent Stem Cells/
9	exp Stromal Cells/
10	stem cell research/
11	(advanced-therapy medicinal product$ or advanced therapy medicinal product$ or atmp$).tw.
12	regenerative medicine$.tw.
13	advanced therap$.tw.
14	(gene-therapy medicinal product$ or gene therapy medicinal product$ or gtmp$).tw.
15	(cell-therapy medicinal product$ or cell therapy medicinal product$ or ctmp$).tw.
16	(tissue engineered product$ or tissue-engineered product$ or tep or teps).tw.
17	(regenerative medicine advanced therap$ or rmat$).tw.
18	"human cells, tissues, and cellular and tissue-based products (HCT/Ps)".tw.
19	"human cells, tissues, and cellular and tissue-based product (HCT/P)".tw.
20	regenerative therap$.tw.
21	("cell and gene therapy product" or "cell and gene therapy products" or cgtp or cgtps).tw.
22	or/1-21
23	(Tisagenlecleucel or Kymriah or cart 19 or cart19 or "ctl 019" or ctl019).tw.
24	(Axicabtagene ciloleucel or Yescarta or kte c19 or ktec19).tw.
25	(Voretigene neparvovec-rzyl or Voretigene neparvovec?rzyl or Luxturna).tw.
26	(Talimogene laherparepvec or Imlygic or oncovex or t vec).tw.
27	(Strimvelis or gsk 2696273 or gsk2696273).tw.
28	Holoclar.tw.
29	MACI.tw.
30	(Provenge or sipuleucel t or apc 8015 or apc8015).tw.
31	(Glybera or alipogene tiparvovec or "amt 011" or amt011 or aav1 lpls447x).tw.
32	Zalmoxis.tw.
33	Spherox.tw.
34	or/23-33
35	22 or 34
**Patient, carer and public filter (adapted SIGN Patient Issues filter)**	
36	((patient$ or consumer$ or public) adj2 (decisi$ or decid$)).ti,ab.
37	"Patient Acceptance of Health Care"/
38	exp attitude to health/
39	Patient Preference/
40	"patient satisfaction".ti.
41	exp health education/
42	patient education as topic/
43	health knowledge, attitudes, practice/
44	"informed choice".ti,ab.
45	"shared decision making".ti,ab.
46	("focus group" adj3 (patient$ or parent$ or famil$ or spouse$ or public)).ti,ab.
47	Consumer Advocacy/
48	patient advocacy/
49	exp professional-patient relations/

50	((patient$ or consumer$ or parent$ or famil$ or spouse$ or carer$ or public) adj (attitude$ or involvement or desir$ or perspective$ or activation or view$ or preference$ or experience$ or knowledge$ or understand$ or awareness$)).ti,ab.
51	exp decision making/
52	exp communication/
53	vignette*.ti,ab.
54	"focus group$".ti,ab.
55	focus groups/
56	exp empirical research/
57	narration/
58	(meta-ethnography or metaethnography).ti,ab.
59	grounded theor*.ti,ab.
60	hermeneutic.ti,ab.
61	(inductive adj2 (analys* or grounded or reasoning)).ti,ab.
62	(ethnograph* or ethnological or ethnomethodol* or ethnonursing research).ti,ab.
63	qualitative.ti.
64	exp qualitative research/
65	(qualitative adj (research or stud* or data)).ab.
66	exp Community Participation/
67	Public Opinion/
68	or/36-67
69	35 and 68
70	limit 69 to yr="2009 -Current"

**Supplementary Table 3. Data extraction form**	
Study title	
Authors	
Year of Publication	
Study type	
Aim of study	
Time period/duration of study	
Country of study	
Source of funding	
Participant group studied	
Number of participants	
Age range	
Gender	
Socioeconomic Status	
Educational level	
Current health status	
Type of therapy being studied	
Standard care	
Results (levels of knowledge and awareness; views; opinions; expectations; experiences; factors influencing any of the foregoing)	
Author conclusions	
Study quality	
Any other observations	

Supplementary Table 4. Characteristics and critical appraisal of included studies

Author	Country	Type of therapy	Participants	Condition	Sample size	Age range (years)	Design/method	Critical appraisal
Aked et al. (2017)[4]	Sweden	Stem cell	Patients	Ischaemic stroke	84 (54 males, 30 females)	20-75; median 68	Quantitative questionnaire	• Response rate 78%<br>• Sample size <100<br>• 91% had no/slight disability (Modified rankin scale) (potential recruitment bias)<br>• Detailed information on socioeconomic status<br>• Funding sources reported (non-pharmaceutical, charities). Authors stated that there were no competing financial interests.
Allum et al. (2017)[5]	Europe; USA; Canada	Stem cell	Public	Not applicable	25,000 Europeans, 2,000 Canadians, 1,200 US citizens	≥18	Re-analysis of existing survey data	• Sample size >100<br>• Detailed information about the surveys<br>• Detailed information on data analysis<br>• Funding sources reported (non-pharmaceutical). Funder was stated to have no role in the project. Authors declared no competing interests.
Benjaminy et al. (2014)[6]	Canada	Gene therapy	Patients; clinicians; patient advocates	Choroideremia	41 (20 male patients, 15 clinicians, 6 patient advocates)	Patients >18	Semi-structured interviews	• Response rate unreported<br>• Some patients were recruited via patient advocacy groups<br>• Limited information on participant socioeconomic status<br>• Detailed information on data collection and analysis<br>• Unclear whether saturation was achieved<br>• Member checking of data analysis was conducted<br>• Funding sources reported (non-pharmaceutical, charities). Authors declared no competing interests.
Blendon et al. (2016)[7]	US	Gene therapy	Public	Not applicable	Not available	Not available	Review of public opinion polls	• Data from 17 polls was reviewed<br>• Demographic information not available due to study method<br>• Limited information on data collection and analysis provided

Supplementary Table 4. Characteristics and critical appraisal of included studies								
Author	Country	Type of therapy	Participants	Condition	Sample size	Age range (years)	Design/method	Critical appraisal
								• Funding source reported (non-pharmaceutical). Authors declared no competing interests.
Bubela et al. (2012)[8]	Canada	Stem cell	Media (newspaper articles)	Not applicable	13,249 newspaper articles, 3,404 clinical trials		Word frequency analysis	• Extensive search of databases<br>• Detailed information on data collection and analysis<br>• Funding sources reported (non-pharmaceutical). Authors declared no competing interests.
Chung et al. (2014)[9]	Korea	Stem cells	Patients; carers	Parkinson's disease	963 patients, 267 carers<br><br>580 male patients, 383 female patients, 143 male carers, 124 female carers	Mean: 70 patients, 65 carers	Questionnaire	• Response rate unreported<br>• Sample size >100<br>• Detailed demographic information provided<br>• Detailed information on data analysis<br>• Funding source unreported. Authors declared no competing interests.
Clover et al. (2012)[10]	Ireland	Stem cell/tissue engineering	Patients	Burns	279 (142 males, 137 females)<br><br>(139 plastic surgery patients and 140 nonplastic surgery patients)	18-89	Quantitative questionnaire	• Response rate 93%<br>• Sample size >100<br>• Modified version of a previously validated questionnaire<br>• Ethnicity, educational and financial status unreported<br>• Funding source unreported. Authors declared no competing interests.
Cunningham (2018)[11]	UK	Stem cell	Patients; carers	Stroke	66 (31 male, 22 female stroke patients; 6 female, 7 male carers)	Patients: 40-75;<br>Carers: Unspecified	Focus group (conversation café)	• Response rate unreported<br>• Sample size appears to be adequate<br>• Ethnicity, educational and financial status unreported<br>• Funding source unclear ("sponsored by trial recruiters"). No statement on competing interest was provided.
Dasgupta et al. (2014)[12]	USA	Stem cell	Patients	Not available	26 (10 males, 16 females)	20-81 (Mean 53.8)	Focus group	• Response rate unreported<br>• Sampling appears to be convenience rather than purposive

Supplementary Table 4. Characteristics and critical appraisal of included studies

Author	Country	Type of therapy	Participants	Condition	Sample size	Age range (years)	Design/method	Critical appraisal
								• Information about health conditions not collected<br>• Unclear if saturation was achieved<br>• Detailed demographic information provided<br>• Funding source reported (non-pharmaceutical). No statement on competing interest was provided.
Eijkholt et al. (2012)[13]	Canada	Stem cell	Friends and family; patients	Spinal cord injury	50 (9 family and friends; 41 HCPs)	Not reported	Focus groups and interviews	• Response rate unreported<br>• Small sample of family/friend<br>• Patients not included<br>• Very limited information on participant characteristics<br>• Details of the data analysis not provided<br>• Funding source reported (non-pharmaceutical). Authors declared no competing interests
Einsiedel et al. (2009)[14]	Canada	Stem cell	Patients; public; caregivers	Not specified	76	Not reported	Focus groups	• Response rate unreported<br>• Detailed information on recruitment, data collection and analysis<br>• Limited information on demographics provided<br>• Unclear if data saturation was achieved<br>• Funding source reported (non-pharmaceutical). No statement on competing interests was provided.
Evans et al. (2011)[15]	USA	Stem cell	Public	Not applicable	2,295 cases	Not reported	Quantitative questionnaire	• Response rate unreported<br>• No information on recruitment process<br>• Sample size >100<br>• No information on participants' demographic characteristics<br>• Detailed information on data analysis<br>• Funding source unreported. Authors declared no competing interests.
Hodges et al. (2012)[16]	Australia	Stem cell	Patients (pregnant women)	General and 15 specific disorders	150	18-40+ (Modal age 26-30)	Quantitative questionnaire	• Response rate 80%<br>• Questionnaire was initially piloted<br>• Sample size >100

Supplementary Table 4. Characteristics and critical appraisal of included studies								
Author	Country	Type of therapy	Participants	Condition	Sample size	Age range (years)	Design/method	Critical appraisal
								• Detailed information on demographics provided<br>• Most of the participants were well educated (potential selection bias)<br>• Funding source reported (non-pharmaceutical). No statement on competing interests was provided.
Horch et al. (2016)[17]	Canada	Stem cell	Public (firefighters)	Not applicable (Discussed use for burns)	149 (144 males, 5 females)	24-63 (mean 41)	Mixed methods; quantitative online survey followed by a qualitative semi-structured interview	• Survey response rate 14%<br>• Sample size >100<br>• Detailed information on demographics provided<br>• Funding sources reported (non-pharmaceutical). Funders' role not specified. No statement on competing interests was provided.
Hudson et al. (2011)[18]	Europe	Gene therapy	Public	Not applicable	Approximately 25,000	Not reported	Quantitative survey	• Response rate unreported<br>• Sample size >100<br>• No information on participants' demographic characteristics<br>• Limited information on data analysis<br>• Funding source unreported. No statement on competing interests was provided.
Jacob et al. (2015)[19]	Canada; USA	Stem cell	Patients	Individuals with spinal cord injury (ISCIs)	18 ISCIs (15 males, 3 females)	20 – 76+	Qualitative interviews	• Response rate unreported<br>• Detailed information was provided on patient characteristics, recruitment, data collection and analysis<br>• Unclear if data saturation was achieved<br>• Funding source reported (non-pharmaceutical). Authors declared no competing interests.
Jannetta et al. (2010)[20]	UK	Gene therapy	Patients	Cystic fibrosis	12 (7 males, 5 females)	19-53	Qualitative interviews	• Response rate 29%*<br>• Detailed information was provided on methodological approach, recruitment, data collection and analysis<br>• Funding source unreported (PhD thesis)
Kim et al. (2013)[21]	South	Stem cell	Patients	Ischaemic	250 (175	23-90 (Mean	Surveys and brief	• Survey response rate 80%

Supplementary Table 4. Characteristics and critical appraisal of included studies								
Author	Country	Type of therapy	Participants	Condition	Sample size	Age range (years)	Design/method	Critical appraisal
	Korea			stroke	males, 75 females)	63)	qualitative interviews	• Sample size >100<br>• Detailed information on demographics provided<br>• Information was not provided on the aim and findings of the interviews<br>• Funding source reported (non-pharmaceutical). Authors declared no competing financial interests.
King et al. (2010)[22]	USA	Stem cell	Patient; Public; HCPs	HIV/AIDs	47 (16 religious leaders, 8 HCPs, 12 patients; 11 community workers)<br><br>23 males, 24 females	Mean range 39-52	Focus groups and surveys	• Response rate unreported<br>• Detailed information on recruitment, data collection and analysis<br>• Detailed information on demographics provided<br>• Unclear if data saturation was achieved<br>• Funding source reported (non-pharmaceutical). No statement on competing interests was provided.
King et al. (2018)[23]	Scotland	Stem cell	Patient; Public	Patients had conditions such as thalassaemia and myelodysplasia	15 interviewees, size of focus groups unreported	Unreported	Ethnographic study; 15 interviews and 12 focus groups	• Response rate unreported<br>• Very limited information on participant characteristics<br>• Detailed information on methodological approach<br>• Limited information on the qualitative analysis provided<br>• Funding source reported (non-pharmaceutical). No statement on competing interests was provided.
Nelissen et al. (2016)[24]	Belgium	Stem cell	Patients; public	Cancer	2008 (621 patients with cancer, 1387 non-diagnosed public).<br>602 males, 1406 females	16-88 (75% between 21 and 60)	Cross sectional survey	• Sample size >100<br>• Detailed information on demographics provided<br>• Detailed information on the qualitative analysis provided<br>• Funding source reported (non-pharmaceutical). Authors declare no conflict of interest.
Nelles et al. (2015)[25]	Germany	Gene therapy	Patients	Genetic eye disease (RPE65	5 (1 male, 4 females)	19-28	Quantitative questionnaires and qualitative	• Very small sample size*<br>• Response rate 55.5%<br>• Ethnicity, educational and financial

Supplementary Table 4. Characteristics and critical appraisal of included studies								
Author	Country	Type of therapy	Participants	Condition	Sample size	Age range (years)	Design/method	Critical appraisal
				deficiency)			interviews	status unreported*<br>• Limited information on the qualitative analysis conducted<br>• Funding source unreported. Authors declare no conflict of interest.
Nisbet et al. (2014)[26]	USA	Stem cell	Public	Not applicable	Not available	Not available	Review of opinion polls	• Data from a number of polls was reviewed<br>• Demographic information not available due to study method<br>• Limited information on data collection and analysis provided<br>• Funding source unreported. No statement on competing interests was provided.
Peay et al. (2018)[27]	USA	Gene therapy	Patients; carers	Duchenne Muscular Dystrophy	6 adult patients with DMD, 17 parents of individuals with DMD	Adult patients 21–26, individuals represented by parents 5–32.	Qualitative interviews	• Recruitment through advocacy organisation<br>• Response rate unreported<br>• Small patient sample size*<br>• Limited information on patients' socio-economic status*<br>• Detailed information on data collection and analysis<br>• Patient advocacy charity and pharmaceutical funding stated.<br>• Funders were not involved in data collection and analysis but were involved in interpretation and generation of conclusions. Authors declared no conflicts of interest.
Rachul et al. (2015)[28]	USA; Canada	Stem cell	Public	Not applicable	Not available	Not available	Qualitative analysis of news media articles and readers' comments	• Although five sport websites and two newspaper websites were searched, study populations may not fully represent sampling population (potential selection bias)<br>• Demographic information not available due to study method<br>• Detailed information on data collection and analysis provided

Supplementary Table 4. Characteristics and critical appraisal of included studies								
Author	Country	Type of therapy	Participants	Condition	Sample size	Age range (years)	Design/method	Critical appraisal
								• Funding source reported (non-pharmaceutical). Authors declare no conflict of interest.
Robillard et al. (2013)[29]	Canada	Gene therapy	Public	Not applicable	Not available	Not available	Content analysis of social media platform (Yahoo! Answers)	• Difficult ascertaining whether study population represents sampling population (potential selection bias)<br>• Demographic information not available due to study method<br>• Framing of questions might have been leading (potential response bias)<br>• Interactions from only one social media platform was analysed (potential selection bias)<br>• Detailed information on data collection and analysis provided<br>• Funding source reported (non-pharmaceutical). Authors declare no conflict of interest.
Robillard et al. (2014)[30]	USA; Canada	Gene therapy	Public	Not applicable	467 complete and valid responses out of 560. Females 56%	19-69 (56% were 19-29)	Online survey	• Surveys were validated<br>• Final percentage analysed 83%<br>• Methods used by the private company for recruitment unclear<br>• Detailed information was provided on data analysis<br>• Younger age groups overrepresented<br>• Funding source reported (non-pharmaceutical). Authors declare no conflict of interest.
Shineha et al. (2018)[31]	Japan	Stem cell	Public; scientists	Not applicable	3275 valid responses (2,160 public; 1,115 scientists)	Not available	Quantitative questionnaire	• Response rate 22.1% for scientists. Rate for general public unreported.<br>• Limited participant demographic information<br>• Limited information on data analysis<br>• Funding source reported (non-pharmaceutical). Authors declare no conflict of interest.
Sipp D. (2017)[32]	USA	Stem cell	Public	Not applicable	400 responses randomly	Not available	Content analysis	• Difficult ascertaining whether study population represents sampling

Supplementary Table 4. Characteristics and critical appraisal of included studies								
Author	Country	Type of therapy	Participants	Condition	Sample size	Age range (years)	Design/method	Critical appraisal
					sampled from a set of 6962			population (potential selection bias)<br>• Demographic information not available due to study method<br>• Framing of questions might have been leading (potential response bias)<br>• Limited information on data analysis<br>• Funding source reported (non-pharmaceutical). Authors declared no competing interests.
Stewart et al. (2015)[33]	UK	Stem cell	Patients	Plastic surgery	50 (15 males, 35 females)	22-88 (Mean age 45.4)	Qualitative interviews and questionnaire	• Response rate unreported<br>• Limited information about socio-economic status of participants<br>• Limited information on questionnaire development<br>• Limited information on data analysis<br>• Funding and conflict of interest were stated as 'none'.
Strong H et al. (2017)[34]	USA	Gene therapy	Patients	Sickle cell	42 (20 males, 22 females)	18-58 (mean 27)	Focus groups	• Response rate 54%<br>• Detailed information on demographics provided<br>• Detailed information on data collection and analysis provided<br>• Funding sources reported (non-pharmaceutical). Funders not involved in the project. Authors declared no competing interests.
Tanner et al. (2017)[35]	Australia	Stem cell	Patients; carers; clinicians; patient advocates	Up to eight diverse conditions	71 (Patients and/or carers who travelled for overseas treatment = 24; Patients contemplating travelling = 27; HCPs/ scientists = 20)	Unreported	Qualitative interviews	• Very limited information on participants characteristics<br>• Current health status of patients not reported<br>• Response rate unreported<br>• Detailed information on recruitment procedure<br>• Limited information on data analysis<br>• Unclear if saturation was achieved and sample size adequate.<br>• Findings may be less specific due to the diversity of conditions.

Supplementary Table 4. Characteristics and critical appraisal of included studies								
Author	Country	Type of therapy	Participants	Condition	Sample size	Age range (years)	Design/method	Critical appraisal
								• Funding source reported (non-pharmaceutical). Authors declared no competing interests.
Vicsek et al. (2011)[36]	Hungary	Stem cell	Media newspaper articles; public	Not applicable	326 articles, 56 focus group participants	25-60	Qualitative content analysis and focus groups	• Response rate (focus group) unreported<br>• Limited participant demographic information<br>• Limited information on data analysis<br>• Unclear if saturation was achieved and sample size adequate.<br>• Funding sources reported (non-pharmaceutical). Funders' role not specified. No statement on competing interests was provided.
Wang et al. (2017)[37]	China	Gene therapy	Public; clinicians	Not applicable	13,201 valid responses (11,036 general public, 2,165 clinicians)<br><br>Females (44.2% clinicians, 42.0% public)	18-50	Online survey	• Sample size >100<br>• Completion rate 97.3%<br>• Detailed information on demographics provided<br>• Recruitment done through social media, personal promotion (potential selection bias)<br>• Funding sources reported (non-pharmaceutical). Authors declared no competing interests.
Wright et al. (2016)[38]	UK	Stem cell	Patients	Possible post-obstetric incontinence	70	16-50	Quantitative questionnaire with some free text response options	• Response rate 92%<br>• Sample size <100<br>• Ethnicity, educational and financial status unreported<br>• Funding sources reported (non-pharmaceutical). Authors declared no competing interests.

References

1. FDA. What Is Gene Therapy? How Does It Work? (US Food and Drugs Administration).
2. EMA. Advanced therapy medicinal products: Overview (European Medicines Agency).
3. Faulkner, A., Geesink, I., Kent, J. & FitzPatrick, D. Human tissue engineered products--drugs or devices? *BMJ (Clinical research ed.)* **326**, 1159-1160 (2003).
4. Aked, J.*, et al.* Attitudes to Stem Cell Therapy among Ischemic Stroke Survivors in the Lund Stroke Recovery Study. *Stem Cells and Development* **26**, 566-572 (2017).
5. Allum, N.*, et al.* Religion and the public ethics of stem-cell research: Attitudes in Europe, Canada and the United States. *PloS one* **12 (4) (no pagination)**(2017).
6. Benjaminy, S., Macdonald, I. & Bubela, T. "Is a cure in my sight?" Multi-stakeholder perspectives on phase I choroideremia gene transfer clinical trials. *Genetics in Medicine* **16**, 379-385 (2014).
7. Blendon, R.J., Gorski, M.T. & Benson, J.M. The Public and the Gene-Editing Revolution. *The New England journal of medicine* **374**, 1406-1411 (2016).
8. Bubela, T., Li, M.D., Hafez, M., Bieber, M. & Atkins, H. Is belief larger than fact: expectations, optimism and reality for translational stem cell research. *BMC Med* **10**, 133 (2012).
9. Chung, S.J., Koh, S.B., Ju, Y.-S. & Kim, J.W. Nationwide Survey of Patient Knowledge and Attitudes towards Human Experimentation Using Stem Cells or Bee Venom Acupuncture for Parkinson's Disease. *JMD* **7**, 84-91 (2014).
10. Clover, A.J., O'Neill, B.L. & Kumar, A.H. Analysis of attitudes toward the source of progenitor cells in tissue-engineered products for use in burns compared with other disease states. *Wound Repair & Regeneration* **20**, 311-316 (2012).
11. Cunningham, N.A., Abhyankar, P., Cowie, J., Galinsky, J. & Methven, K. Regenerative medicine: Stroke survivor and carer views and motivations towards a proposed stem cell clinical trial using placebo neurosurgery. *Health Expect* **21**, 367-378 (2018).
12. Dasgupta, I.*, et al.* Patients' attitudes toward the donation of biological materials for the derivation of induced pluripotent stem cells. *Cell Stem Cell* **14**, 9-12 (2014).
13. Eijkholt, M., Kwon, B.K., Mizgalewicz, A. & Illes, J. Decision-making in stem cell trials for spinal cord injury: the role of networks and peers. *Regen* **7**, 513-522 (2012).
14. Einsiedel, E.*, et al.* Diversity in public views toward stem cell sources and policies. *Stem cell rev* **5**, 102-107 (2009).
15. Evans, M.D. & Kelley, J. US attitudes toward human embryonic stem cell research. *Nature Biotechnology* **29**, 484-488 (2011).
16. Hodges, R.J., Bardien, N. & Wallace, E. Acceptability of stem cell therapy by pregnant women. *Birth (Berkeley, Calif.)* **39**, 91-97 (2012).
17. Horch, J.D.*, et al.* Firefighter willingness to participate in a stem cell clinical trial for burns: A mixed methods study. *Burns* **42**, 1740-1750 (2016).
18. Hudson, J. & Orviska, M. European attitudes to gene therapy and pharmacogenetics. *Drug Discov Today* **16**, 843-847 (2011).
19. Jacob, K.J., Kwon, B.K., Lo, C., Snyder, J. & Illes, J. Perspectives on strategies and challenges in the conversation about stem cells for spinal cord injury. *Spinal Cord* **53**, 811-815 (2015).
20. Jannetta, E.E., Cochrane, S.C., Morris, P.G., Dewar, M.H. & Innes, J.A. A qualitative study of cystic fibrosis (CF) patients' expectations of gene therapy. *Journal of Cystic Fibrosis* **1)**, S101 (2010).
21. Kim, Y.S.*, et al.* Fantasies about stem cell therapy in chronic ischemic stroke patients. *Stem Cells Dev* **22**, 31-36 (2013).
22. King, W.D.*, et al.* Pilot assessment of HIV gene therapy-hematopoietic stem cell clinical trial acceptability among minority patients and their advisors. *J Natl Med Assoc* **102**, 1123-1128 (2010).
23. King, E. & Lyall, C. What's in a name: are cultured red blood cells 'natural'? *Sociology of health & illness* **40**, 687-701 (2018).

24. Nelissen, S., Van den Bulck, J., Lemal, M. & Beullens, K. Stem cell research: the role of information seeking and scanning. *Health Info Libr J* **33**, 269-282 (2016).
25. Nelles, M., Stieger, K., Preising, M.N., Kruse, J. & Lorenz, B. Shared decision-making, control preferences and psychological well-being in patients with RPE65 deficiency awaiting experimental gene therapy. *Ophthalmic Res* **54**, 96-102 (2015).
26. Nisbet, M.C. & Becker, A.B. Public opinion about stem cell research, 2002 to 2010. *Public Opinion Quarterly* **78**, 1003-1022 (2014).
27. Peay, H.*, et al.* Parent and adult patient attitudes about gene therapy as a therapeutic option for duchenne muscular dystrophy. *Value in Health* **21 (Supplement 1)**, S256 (2018).
28. Rachul, C. & Caulfield, T. Gordie Howe's Stem Cell 'Miracle': A Qualitative Analysis of News Coverage and Readers' Comments in Newspapers and Sports Websites. *Stem Cell Reviews and Reports* **11**, 667-675 (2015).
29. Robillard, J.M.*, et al.* Utilizing social media to study information-seeking and ethical issues in gene therapy. *Journal of medical Internet research* **15**, e44 (2013).
30. Robillard, J.M., Roskams-Edris, D., Kuzeljevic, B. & Illes, J. Prevailing public perceptions of the ethics of gene therapy. *Human Gene Therapy* **25**, 740-746 (2014).
31. Shineha, R., Inoue, Y., Ikka, T., Kishimoto, A. & Yashiro, Y. A Comparative Analysis of Attitudes on Communication Toward Stem Cell Research and Regenerative Medicine Between the Public and the Scientific Community. *Stem Cells Transl Med* **7**, 251-257 (2018).
32. Sipp, D. Identity and ownership issues in the regulation of autologous cells. *Regen* **12**, 827-838 (2017).
33. Stewart, C.J.*, et al.* Patient's attitude towards the donation and use of adipose tissue and adipose derived stem cells for research. *Journal of Plastic, Reconstructive and Aesthetic Surgery* **68**, 588-589 (2015).
34. Strong, H.*, et al.* Patient Perspectives on Gene Transfer Therapy for Sickle Cell Disease. [Erratum appears in Adv Ther. 2017 Aug 2;:; PMID: 28770518]. *Adv Ther* **34**, 2007-2021 (2017).
35. Tanner, C., Petersen, A. & Munsie, M. 'No one here's helping me, what do you do?': Addressing patient need for support and advice about stem cell treatments. *Regen* **12**, 791-801 (2017).
36. Vicsek, L. & Gergely, J. Media presentation and public understanding of stem cells and stem cell research in Hungary. *New Genetics and Society* **30**, 1-26 (2011).
37. Wang, J.H.*, et al.* Public Attitudes toward Gene Therapy in China. *Mol Ther Methods Clin Dev* **6**, 40-42 (2017).
38. Wright, B.*, et al.* Women's views on autologous cell-based therapy for post-obstetric incontinence. *Regen* **11**, 169-180 (2016).
