# Supplementary material for: Patient and public perspectives on cell and gene therapies: a systematic review
Source: Nat Commun. 2020 Dec 8;11:6265. doi: 10.1038/s41467-020-20096-1 (PMC7722871; doi:10.1038/s41467-020-20096-1)
Supplement: Supplementary file 3 — Reporting Summary [file 41467_2020_20096_MOESM3_ESM.pdf]

nature research

Corresponding author(s): Olalekan Lee Aiyegbusi

Last updated by author(s): Oct 9, 2020

Reporting Summary

Nature Research wishes to improve the reproducibility of the work that we publish. This form provides structure for consistency and transparency in reporting. For further information on Nature Research policies, see our Editorial Policies and the Editorial Policy Checklist.

Statistics

For all statistical analyses, confirm that the following items are present in the figure legend, table legend, main text, or Methods section.

n/a	Confirmed	
x	☐	The exact sample size (*n*) for each experimental group/condition, given as a discrete number and unit of measurement
x	☐	A statement on whether measurements were taken from distinct samples or whether the same sample was measured repeatedly
x	☐	The statistical test(s) used AND whether they are one- or two-sided<br>*Only common tests should be described solely by name; describe more complex techniques in the Methods section.*
x	☐	A description of all covariates tested
x	☐	A description of any assumptions or corrections, such as tests of normality and adjustment for multiple comparisons
x	☐	A full description of the statistical parameters including central tendency (e.g. means) or other basic estimates (e.g. regression coefficient) AND variation (e.g. standard deviation) or associated estimates of uncertainty (e.g. confidence intervals)
x	☐	For null hypothesis testing, the test statistic (e.g. *F*, *t*, *r*) with confidence intervals, effect sizes, degrees of freedom and *P* value noted<br>*Give P values as exact values whenever suitable.*
x	☐	For Bayesian analysis, information on the choice of priors and Markov chain Monte Carlo settings
x	☐	For hierarchical and complex designs, identification of the appropriate level for tests and full reporting of outcomes
x	☐	Estimates of effect sizes (e.g. Cohen's *d*, Pearson's *r*), indicating how they were calculated

Our web collection on statistics for biologists contains articles on many of the points above.

Software and code

Policy information about availability of computer code

Data collection	No software/tool/algorithm was used for data collection
Data analysis	No software/tool/algorithm was used for data analysis

For manuscripts utilizing custom algorithms or software that are central to the research but not yet described in published literature, software must be made available to editors and reviewers. We strongly encourage code deposition in a community repository (e.g. GitHub). See the Nature Research guidelines for submitting code & software for further information.

Data

Policy information about availability of data

All manuscripts must include a data availability statement. This statement should provide the following information, where applicable:

- Accession codes, unique identifiers, or web links for publicly available datasets
- A list of figures that have associated raw data
- A description of any restrictions on data availability

The authors declare that all data generated or analysed during this study are included in this published article and in Supplementary Table 4. All the publications included in this systematic review are available through open access or personal or institutional journal subscriptions.

Field-specific reporting

Behavioural & social sciences study design

All studies must disclose on these points even when the disclosure is negative.

Study description	This is an analysis of studies which assessed the perspectives of patients and the public on cell and gene therapy. A systematic search was conducted to identify relevant studies. The majority of the studies included were qualitative. However, some also utilized survey methods.
Research sample	This was a systematic review, no primary data collection was performed
Sampling strategy	This was a systematic review, no primary data collection was performed
Data collection	This was a systematic review, no primary data collection was performed
Timing	This was a systematic review, no primary data collection was performed
Data exclusions	Exclusion criteria<br>- Narrative reviews, commentaries, opinion pieces and letters that do not report primary findings.<br>- Studies focused exclusively on the perspectives of healthcare professionals and or scientists<br>- Studies focused entirely on embryonic stem cell therapy or germline gene therapy (due to the complex legal and ethical issues associated with these therapies)<br>- Studies reporting hematopoietic stem cell transplants (as this type of treatment is no longer novel)
Non-participation	This was a systematic review, no primary data collection was performed
Randomization	This was a systematic review, no primary data collection was performed

Reporting for specific materials, systems and methods

We require information from authors about some types of materials, experimental systems and methods used in many studies. Here, indicate whether each material, system or method listed is relevant to your study. If you are not sure if a list item applies to your research, read the appropriate section before selecting a response.

Materials & experimental systems

n/a	Involved in the study	
☒	☐	Antibodies
☒	☐	Eukaryotic cell lines
☒	☐	Palaeontology and archaeology
☒	☐	Animals and other organisms
☒	☐	Human research participants
☒	☐	Clinical data
☒	☐	Dual use research of concern

Methods

n/a	Involved in the study	
☒	☐	ChIP-seq
☒	☐	Flow cytometry
☒	☐	MRI-based neuroimaging
