# Supplementary material for: Patient and public perspectives on cell and gene therapies: a systematic review
Source: Nat Commun. 2020 Dec 8;11:6265. doi: 10.1038/s41467-020-20096-1 (PMC7722871; doi:10.1038/s41467-020-20096-1)
Supplement: Supplementary file 2 — Peer Review File [file 41467_2020_20096_MOESM2_ESM.pdf]

Editorial Note: This manuscript has been previously reviewed at another journal that is not operating a transparent peer review scheme. This document only contains reviewer comments and rebuttal letters for versions considered at *Nature Communications*. Mentions of prior referee reports have been redacted.
